# Management of Hyperglycaemia by Ethyl Acetate Extract of *Balanites aegyptiaca* (Desert Date)

**DOI:** 10.3390/molecules200814425

**Published:** 2015-08-07

**Authors:** Abdulrahman L. Al-Malki, Elie K. Barbour, Kalid O. Abulnaja, Said S. Moselhy

**Affiliations:** 1 Department of Biochemistry, Faculty of Science, King Abdulaziz University, P.O. Box 80205, Jeddah 22254, Saudi Arabia; E-Mails: kabualnaja@kau.edu.sa (K.O.A.); moselhy6@hotmail.com (S.S.M.); 2 Bioactive Natural Products Research Group, Faculty of Science, King Abdulaziz University, P.O. Box 80205, Jeddah 22254, Saudi Arabia; E-Mail: kabualnaja@kau.edu.sa; 3 Experimental Biochemistry Unit, King Fahd Medical Research Center, King Abdulaziz University, P.O. Box 80205, Jeddah 22254, Saudi Arabia; 4 Adjunct to Biochemistry Department, Faculty of Science, King Abdulaziz University, P.O. Box 80205, Jeddah 22254, Saudi Arabia; 5 Agriculture Department, Faculty of Agricultural and Food Sciences, American University of Beirut, P.O. Box 11-0236, Beirut 1107 2020, Lebanon; 6 Department of Biochemistry, Faculty of Science, Ain Shams University, Cairo 11566, Egypt

**Keywords:** *Balanites aegyptiaca*, antidiabetic, ethyl acetate extract, inflammatory cytokines

## Abstract

Reactive oxygen species play a significant role in the pathogenesis of retinopathy in diabetes patients. The current study aimed to assess the effect of ethyl acetate extract (EAE) from *Balanites aegyptiaca* (10, 25 or 50 mg/kg b.w.) in experimental diabetic rats. To achieve this aim, five groups of male rats were included: control, diabetic, and diabetic rats treated with 10, 25, and 50 µg/kg b.w. of EAE for eight weeks. Our results suggests a protective role of EAE against oxidative stress induced by streptozocine. EAE treatment produced a reduction in blood glucose levels, HbA1c, malondialdehyde and vascular endothelial growth factor (VEGF) in diabetic retina (*p* < 0.001), as well as an enhancement in antioxidant capacity against streptozocine-induced oxidative stress. Tumor necrosis factor alpha (TNF-α), interleukin (IL-1β) and vascular endothelial growth factor (VEGF) were significantly reduced in diabetic rats treated with EAE, compared with untreated diabetic rats. Analysis of EAE by GC-MS indicated the presence of β-sistosterol. Overall, EAE modulates oxidative stress induced by streptozocine and enhances antioxidant activity, which may provide additional endothelial protection in retina of diabetic rats. These results hold great promise in the management of diabetic complications.

## 1. Introduction

Diabetes mellitus is a chronic disease characterized by sustained hyperglycemia. It is a high risk factor for the development of many complications like nephropathy, retinopathy and production of free radicals [1]. Additionally, excessive glucose can result in the production of highly active intermediate compounds, leading to non-functional proteins. This can result in the formation of protein-adducts which promote the formation of the highly modified advanced glycation end products (AGEs) [2]. It was proven that there is a close relationship between the degree of free radical generation and the extent of inflammation in the pathophysiology of diabetics [3]. The prevention of diabetic complications in the early stages of the disease is of greatest significance. Natural products or their derivatives can act as antioxidants that possess integrative and complementary medicinal properties in the protection against these complications [4].

Diabetic retinopathy is a leading cause of acquired blindness in adults. The microvasculature of the retina is damaged, the blood vessels swell and leak fluid if not prevented, and new vessels grow, ultimately leading to the detachment of the retina [5,6,7]. The prevalence of diabetic retinopathy is increasing worldwide due to the prolonged survival of diabetic patients. 

It was suggested that hyperglycemia plays a major role in the pathogenesis of retinopathy. The most important mechanisms are the oxidative stress pathway [8,9], polyol pathway activity [10], formation of advanced glycation end-products (AGEs) [11], activation of protein kinase C isoforms [12] and augmentation of the hexosamine pathway flux [13]. 

As a rich source for the discovery of different drugs such as phytomedicines, medicinal plants have been widely studied by the [14]. Among the known naturally occurring antioxidants, phenolic and epicatichin compounds are the most widely studied. *Balanites aegyptiaca* Del. (Zygophyllaceae), also known as “desert date”, is a spiny tree of up to l0 m in height, distributed in Africa and South Asia. The fruits of this plant are used as folk medicine in the treatment of various diseases such as intestinal worm infections, wound healing, syphilis, dysentery, constipation, diarrhea and fever. The fruits of *Balanites aegyptiaca* is known to contain a wide variety of compounds, which show a wide range of biological and pharmacological properties such as antioxidant, anti-inflammatory, antimicrobial and cytotoxic activities [14]. A preliminary study was performed in our lab to investigate the toxicity of these extracts in mice, including body weight, liver, kidney and heart function. No significant changes were observed. 

There is no adequate data reporting the effects of *Balanites aegyptiaca* extract on the progression and development of diabetes. In this work we evaluated the chemical profile and antidiabetic potential of ethyl acetate extracts (EAE) of *Balanites aegyptiaca* fruits. Both anti-inflammatory and antioxidant effects of the fruits were examined to evaluate their efficacy as a drug to attenuate and/or prevent diabetic retinopathy in rats. 

## 2. Results

Table 1 shows the GC-MS analysis of EAE of fruits of *B. aegyptiaca* revealed the presence of vanillic acid (26.58%) with the molecular formula C_8_H_8_O_4_, and MW 312, syringic acid (24.08%) with molecular formula C_9_H_10_O_5_, and MW 342 and, β-sitosterol (23.94%) with molecular formula C_29_H_50_O, and MW of 414. 

**Table 1:** GC-MS analysis of *Balanites aegyptiaca* extract.

Name of Compound	MW	Peak Area in %	RT	S. No.
Vanillic acid	312	26.58	15.02	1
unknown	--	8.340	15.30	2
unknown	--	2.220	15.41	3
unknown	--	3.210	15.56	4
unknown	--	2.870	15.73	5
Syringic acid	342	24.08	18.04	6
β-sitosterol	414	23.94	24.50	7

Rats injected with STZ experienced a significant elevation in blood glucose level compared with the control group (*p* < 0.001), while treatment with EAE of *B. aegyptiaca* at 10, 25 and 50 mg /kg b.w.; resulted in a significant reduction in blood glucose compared with the untreated diabetic animals (*p* < 0.05, *p* < 0.05, *p* < 0.001, respectively in Table 2). The observed effects were dose dependent, but did not return to normal values after the treatment. HbA1c was also significantly increased (*p* < 0.001) in the diabetic rats compared with control group. However, treatment of animals with EAE of *B. aegyptiaca* at 10, 25 and 50 mg /kg b.w. reduced HbA1cin a dose dependent manner (*p* < 0.01, *p* < 0.01, *p* < 0.001, respectively in Table 1). Our results showed that the levels of serum TNF-α and IL-1β were significantly elevated in diabetic rats compared with the control ones. The elevated levels of TNF-α (*p* < 0.01, *p* < 0.01, *p* < 0.001) and IL-1β (*p* < 0.05, *p* < 0.01, *p* < 0.001) were significantly and dose-dependently reduced as a result of EAE administration. The highest effect was found in the highest dose of EAE.

The cellular antioxidant capacity as measured by GSH decreased (*p* < 0.001) and the activities of the enzymes SOD and glutathione peroxidase were significantly decreased (*p* < 0.001) in retina in STZ injected rats. In addition, elevation of malondialdehyde (MDA) levels was seen in the diabetic group compared with the control group (*p* < 0.001). Supplementation of EAE at 10, 25 and 50 mg /kg b.w resulted in a significant increase in the activity of these enzymes (Table 3) with concomitant reduction of MDA. The enhancement of enzyme activity was found to be dose dependent. 

**Table 2:** Glycated hemoglobin and serum IL-1β and TNF-α levels in the diabetic rats dosed with the extracts of *B. aegyptiaca* and control group (Mean ± SD).

Groups	HA_1_C (%)	Glucose (mg/dL)	TNF-α (ng/dL)	IL-1β (ng/dL)
Control Mean ± SD	4.2 ± 0.51	92.5 ± 3.8	0.13 ± 0.013	220 ± 38
Diabetic Mean ± SD	7.4 ± 0.82 ^a,b^	340 ± 15.8 ^a,b^	2.54 ± 0.122 ^a,b^	902 ± 56 ^a,b^
Diabetic+ BE (10 mg) Mean ± SD	6.6 ± 0.63 ^a,b^	280 ± 13 ^a,b^	0.92 ± 0.05 ^a,b^	637 ± 25 ^a,b^
Diabetic+ BE (25 mg) Mean ± SD	5.8 ± 0.55 ^a,b^	240 ± 10.7 ^a,b^	0.82 ± 0.05 ^a,b^	430 ± 33 ^a,b^
Diabetic+ BE (50 mg) Mean ± SD	5.7 ± 0.60 ^a,b^	140 ± 2.1 ^a^	0.88 ± 0.04 ^a,b^	312 ± 21 ^a^

^a^*: p* value <0.05 comparing with control, ^b^: *p* value <0.05 comparing with diabetic.

**Table 3:** Lipid peroxidation level (MDA) reduced glutathione and antioxidant enzymes activities: glutathione S-transferase (GST), superoxide dismutase (SOD) and glutathione peroxidase (GSH-Px) in rats treated with extracts of *B. aegyptiaca* (Mean ± SD) and control group.

Animal Groups	Control	Diabetic	Dia+EAE (10 µg)	Dia+EAE (25 µg)	Dia+EAE (50 µg)
MDA(nmol/mg protein) Mean ± SD	3.5 ± 0.1	13 ± 1.6 ^a^	9 ± 1.1 ^a,b^	5.0 ± 0.8 ^a,b^	4.5 ± 0.5 ^a,b^
GSH( nmol/mg protein) Mean ± SD	88 ± 13.2	43 ± 2.8 ^a^	45 ± 6.0 ^a,b^	52 ± 3 ^a,b^	58 ± 8 ^a,b^
GST( nmol/mg protein) Mean ± SD	19 ± 1.3	4 ± 0.8 ^a^	10 ± 1.0 ^a,b^	11 ± 1.0 ^a,b^	16 ± 2.0 ^a^
SOD (U/ mg protein) Mean ± SD	17.8 ± 1.6	3.1 ± 1.2 ^a^	10.97 ± 1.5 ^a,b^	11.2 ± 1.3 ^a,b^	14.25 ± 1 ^a^
GSH-Px( U /mg protein) Mean ± SD	49.5 ± 4.14	21 ± 4.1 ^a^	31.12 ± 7.5 ^a,b^	29 ± 6.1 ^a,b^	36 ± 9.2 ^a,b^

a: *p* value < 0.05 comparing with control, b: *p* value <0.05 comparing with diabetic.

**Figure 1:**
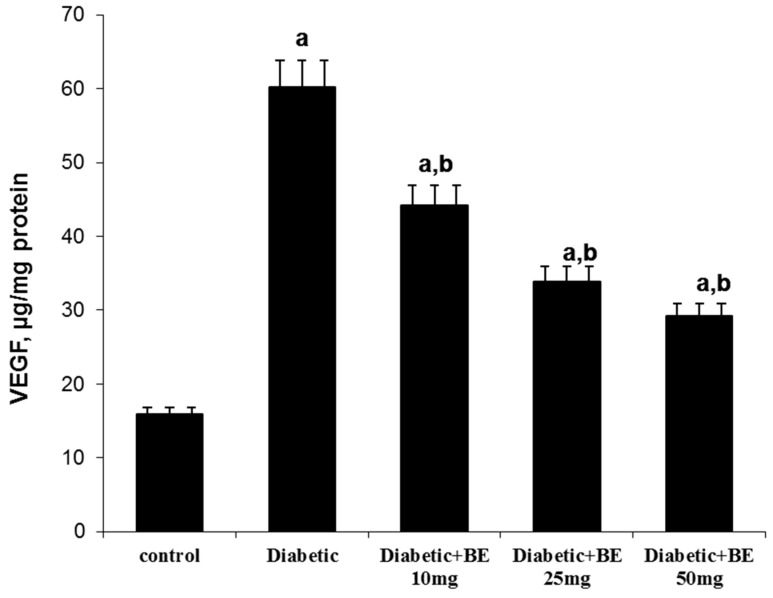
The VEGF level in retina of rats in different groups, control, diabetic and diabetic treated with extracts of *B. aegyptiaca* at different concentrations (mean ± SD). a: Reference *p* value < 0.05 compared with control, b: *p* value < 0.05 compared with diabetic rats.

VEGF plays an important role in the pathogenesis of diabetic retinopathy. The levels of VEGF were measured in the retina of diabetic rats. STZ injection increased VEGF levels, suggesting inflammation in diabetic rats compared with the control rats. Administration of EAE of *B. aegyptiaca* at 10, 25 and 50 mg/kg b.w.; (*p* < 0.01, *p* < 0.01, *p* < 0.001, respectively) resulted in a significant and a dose dependent reduction in VEGF in retina (Figure 1). 

## 3. Discussion

The GC-MS of the EAE revealed the presence of vanillic acid, syringic acid, and β-sitosterol, which are antioxidants. However, Sheikha *et al.* [15] reported that thin layer chromatography analysis of the extracted compound from *B. aegyptiaca* was saponin. While balanitoside belongs to a large family of compounds structurally-related to steroids. 

Our results showed a hypoglycemic effect of EAE of fruits of *B. aegyptiaca* as indicated by a reduction in the levels of fasting blood glucose and glycated hemoglobin in diabetic rats compared with control. The results were dose-dependent and the highest effect was observed at 50 mg/kg b.w. These effects can be attributed to high content of bioactive compounds as suggested by Georg *et al.* [16] and Mansour *et al*. [17]. The pure saponin extracted from the balamite fruit mesocarp, was reported as a hypoglycemic agent when tested on albino rats in different doses. The aqueous extract of the mesocarp of fruits of *B. aegyptiaca* was reported to have antidiabetic effect in streptozotocin-induced diabetic mice [17].

It was found that inflammatory mediators such as TNF-α and IL-1β contribute to the pathogenesis of diabetic retinopathy [18] and significantly higher levels of these markers were found in diabetic *vs.* healthy control subjects [19]. The role of inflammatory cytokines TNF-α and IL-1β in the damage of retinal endothelial cells during the early and late stages of diabetic retinopathy in a rat model with streptozotocin-induced diabetes has been investigated [20]. Diabetic rats treated with the EAE of *B. aegyptiaca* at 10, 25 and 50 mg/kg b.w. showed significantly lower levels of these inflammatory markers compared with untreated diabetic rats. Other studies also reported that antioxidants dose-dependently inhibit inflammatory markers like TNF- α and IL-1β in *in vitro* studies and their release from a human cancer cell line [21,22]. 

In this study, we showed that the retinoprotective effect of EAE of *B. aegyptiaca* is not only attributable to its inhibitory effect on the release of the inflammatory mediators TNF-α and IL-1β, but also to its antioxidant properties. MDA, a product of lipid peroxidation, is increased in the rat retina as a result of diabetic complications. However, we showed that EAE at 10, 25 and 50 mg/kg b.w. significantly reduced MDA formation. In other words, the mechanism of the inhibitory effects, by which EAE protects against lipid peroxidation, may involve radical scavenging. STZ not only initiates lipid peroxidation but also reduces tissue GSH, GSH-px and SOD activities, and this depletion may result from oxidative modification of these proteins as has been reported in previous studies [23,24,25]. The antioxidant activity of EAE may be due to its high content of vanillic acid, syrigic acid and β-siststerol which act as free radical scavenger and enhance antioxidant activity.

Previous studies have shown that VEGF plays a crucial role in the pathogenesis of diabetic retinopathy [26,27]. It was found that retinal VEGF was significantly increased in diabetic rats compared to control rats. However, treatment with EAE at concentrations 10 or 25 or 50 mg/kg b.w. inhibited this elevation in VEGF. Natural antioxidants were found to reduce angiogenesis by interfering with the formation of VEGF receptor complex which may have physiological significance in the management of diabetic retinopathy [28,29]. 

In conclusion, it can be postulated that EAE of *B. aegyptiaca* fruits has potential benefits in the prevention of retinopathy in diabetes via inhibition of free radical production and enhancement of antioxidant potential.

## 4. Experimental Section

### 4.1. Extraction of Balanites aegyptiaca

*Balanites aegyptiaca* fruits were purchased from local markets in Northern Egypt and were characterized at the Botany Department, Faculty of Science, KAU, Saudi Arabia. The fruits were washed, kernels were removed and the fruits were suspended in ethyl acetate (100 g/L) for 24 h. The resulting extract (EAE) was centrifuged for 10 min at 8000× *g*, then deproteinated with isopropanol (ratio of 1:1) and concentrated by rotary evaporation at 4 °C. Identification of the active components of *B. aegyptiaca* was performed by using gas chromatography-mass spectrometry (GC-MS).

### 4.2. Gas Chromatography-Mass Spectrometry Analysis (GC-MS)

Analysis of EAE of *B. aegyptiaca* fruits was carried out by using a Shimadzu GC-MS equipped with silica type DB1 capillary column (30 m × 0.25 mm i.d.), film thickness 0.1 µm. The temperature of injector was 300 °C and the oven temperature was 300 °C. Helium (99.9%) was used as the carrier gas, at a flow rate of 0.90 mL/min. Samples of 2 µL of *B. aegyptiaca* were injected. The compounds were identified using the database of the National Institute of Standards and Technology (NIST) library. 

### 4.3. Animal Experimental Design 

Seven weeks-old male albino rats weighting 120 ± 10 g were obtained from the animal house of King Fahd Medical Research Center (KFMRC), KAU, Jeddah, and housed in stainless steel cages. The animals received a normal diet and water *ad libitum*. The room temperature was 24 ± 2 °C. The handling of animals was done according to the ethical rules approved by King Abdulaziz University. Overnight fasting sixty rats were given a single dose of streptozocine (STZ) at 65 mg/kg b.w.; intraperitoneal, which were previously injected with 80 mg/ kg b.w.; nicotinamide for induction of type 2 diabetes. Rats with fasting blood sugar level above 250 mg/dL after 72 h were considered as diabetic. Diabetic rats were divided into four groups: diabetic untreated, diabetic treated orally by tube feeding with *B. aegyptiaca* EAE (10, 25 and 50 mg/kg b.w./day) for 8 weeks. In addition, fifteen normal rats were considered as negative control. Blood glucose was determined spectrophotometric by commercial kit purchased from Diagnostic Company (Jeddah, Saudi Arabia), and glycated hemoglobin by ELISA kit from MYBIOSOURCE company (San Diego, CA, USA) were determined immediately. Serum was separated, stored at −80 °C for assay of tumor necrosis factor alpha (TNF-α), and interleukin one beta (IL-1β) using commercially available ELISA kits (BioRad, Hemel Hempstead, UK).

### 4.4. Determination of Protein in the Retina 

The right eye of rats was separated, washed with cold phosphate buffered saline (PBS) (pH 6.3) to remove any blood. The tissue was homogenized in a lysis buffer (63 mM, pH 6.8 Tris-HCl; SDS 0.1%; Nonidet P-40 1%; 1 mM-EDTA; 150 mM-sodium chloride; EGTA 5 mM; aprotinin 1 μg/mL; 1 mM phenyl methyl sulphonyl fluoride; and 2 mM benzamidine; 1 mM of sodium florid; SDS 0.1%). The protein level was quantified by using Folin-Ciocalteau reagent (Pierce, Rockford, IL, USA). The supernatant was stored at −80 °C.

### 4.5. Determination of Malondialdhyde (MDA)

The levels of MDA in retina was determined according to the method of Ohkawa [30]. In this assay, a colored complex was produced between the thiobarbituric acid and the products of the lipid peroxidation at 100 °C in acid medium. The absorption was measured UV/visible spectrophotometer at 532 nm (JENWAY, Staffordshire, UK). 

### 4.6. Determination of Reduced (GSH)

The GSH was assayed by the dithionitrobenzoic acid method using a UV/visible spectrophotometer (JENWAY) according to Moron *et al.* [31].

### 4.7. Assay of Superoxide Dismutase (SOD) Activity

The SOD activity was determined as amount of protein which can neutralize half of superoxide radical generated in the presence of EDTA and riboflavin. This volume was equivalent to 1 SOD unit of activity [32].

### 4.8. Determination of Glutathione Peroxidase

The method of Paglia and Valentine [33] was used for the determination of the activity of glutathione peroxidase in the liver homogenateusing kit from BioRad. The activity was expressed as U/mg protein. 

### 4.9. Determination of VEGF

The levels of vascular endothelial growth factor (VEGF) were assayed by ELISA using kits from BioRad. The assay procedures were performed according to the instructions provided with each kit.

### 4.10. Statistical Analysis

All data were analyzed by using the Statistical Package for the Social Sciences (SPSS) software (version 19, IBM, Armonk, New York, NY, USA). The student's *t*-test was used to examine the statistical significance of the differences between the different groups in the experiment. The ANOVA test was performed to find the difference between groups on some variable. The *p* value of < 0.05 was considered significant.
